# Right-side inguinal canal endometriosis at ultrasound: A case report

**DOI:** 10.18502/ijrm.v20i1.10409

**Published:** 2022-02-18

**Authors:** Abolfazl Mehdizadeh, Shahla Chaichian, Shahla Mirgaloybayat, Samaneh Rokhgireh, Kobra Tahermanesh, Maryam Kadivar, Farahnaz Farzaneh

**Affiliations:** ^1^Endometriosis Research Center, Iran University of Medical Sciences, Tehran, Iran.; ^2^Pars Advanced and Minimally Invasive Medical Manners Research Center, Pars Hospital, Tehran, Iran.; ^3^Department of Pathology, Hazrat-e-Rasool Akram General Hospital, Iran University of Medical Sciences, Tehran, Iran.

**Keywords:** Endometriosis, Inguinal, Ultrasound, Case report.

## Abstract

**Background:**

The first case of inguinal endometriosis was described by Cullen. Endometriosis in the round ligament could be in the pelvic or inguinal area and is a rare disease occurring in 0.6% of women. Women with inguinal endometriosis have a painful inguinal mass during menstrual cycles and they mostly have a history of surgery. The right side is more commonly involved in inguinal endometriosis than the left side (90-94%). A history of gynecologic or abdominal surgery is common in women with inguinal endometriosis.

**Case presentation:**

In our case, a 39-yr-old virgin woman presented with localized pain in the right inguinal that had been present for 4 yr. She did not have any history of previous surgery, and abdominal ultrasonography showed a hypoechoic mass with minimal vascularity. Inguinal endometriosis was correctly diagnosed by two expert radiologists preoperatively, and she underwent laparoscopic surgery.

**Conclusion:**

Considering inguinal endometriosis in the differential diagnosis of women with inguinal masses is important, even if there is no history of gynecologic or abdominal surgery.

## 1. Introduction

Endometriosis was first described by Rokitansky in 1860 and it is a common gynecological disease. Endometriosis is characterized by proliferation of functioning endometrial glands and stroma (endometrium) outside the uterine cavity (pelvis or extra pelvic) (1-4).

The prevalence of endometriosis in women of reproductive age is 10-20%, with occurrence mainly in the pelvic peritoneum, pouch of Douglas, fallopian tubes and ovaries. There are many case reports of endometriosis in almost every organ except the spleen. Extra pelvic endometriosis is rare and refers to implants found in the urinary tract and lungs, gastrointestinal tract, bone, episiotomy scars and rarer places such as the umbilicus, surgical scars, inguinal canal, extremities, skin and central nervous system (5-7).

Inguinal endometriosis is rare and often overlooked or misdiagnosed. The most common symptoms are a change of inguinal mass size and inguinal pain during the menstrual cycle (8).

Differential diagnosis of a suspected inguinal mass is important and the causes of inguinal masses include hernia, neuroma, abscess, hydroceles, lymphadenopathy, lymphoma, lipoma and cancer (9).

## 2. Case presentation

A 39-yr-old virgin female gynecologist presented at Hazrat-e Rasoul Hospital, (Tehran, Iran) in December 2020 with a localized pain in the right inguinal area. The pain had started four yr previously and at this point was very tender, especially during the first to fifth days of her menstrual cycle. The participant had no specific medical or abdominal surgery history. She did not have any family history of endometriosis or hernia; she had regular menstruation at 26-day intervals without any abnormal uterine bleeding. Her menstruation period was 3-4 days. Physical examination four yr previously showed a tender mass measuring approximately 1 × 1 cm located on the right, superior to the pubic tubercle. The size of the mass was larger after four yr (3 × 1 cm). The position of the mass remained unchanged and was not related to cough or force.

**Figure 1:**
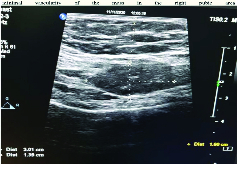
Inguinal ultra-sonographic findings of the inguinal mass.

**Figure 2:**
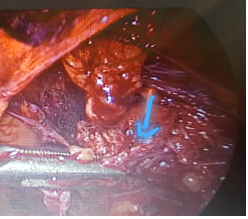
Complete excision of inguinal endometriosis by laparoscopy.

**Figure 3:**
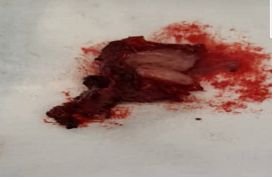
Inguinal endometriosis.

**Figure 4:**
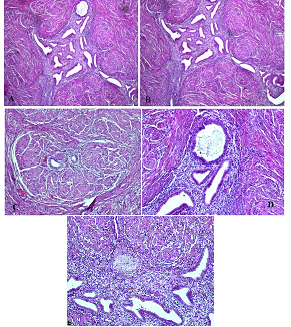
Endometrial gland and stroma surrounded by muscle bundles. A, B, C: H & E staining x200 magnification; D, E: H & E staining x400 magnification.

An ultrasound had been performed four yr before in Tabriz, Azerbaijan, which showed a small inguinal hernia on the right. After four yr, a transabdominal ultrasound was repeated by two expert radiologists and they reported a uterus volume of 36 × 42 × 73 mm^3^ with a normal myometrium; endometrial thickness of 3 mm; and a right ovary of 7 cc and left ovary of 5 cc. The two adnexa were normal. According to an ultrasound of the inguinal canal, there was one hypoechogenic mass with a size of 30 × 14 mm in the right inguinal; a color Doppler showed minimal vascularity and so an inguinal hernia was ruled out (Figure 1). The following tumor markers were measured: cancer antigen 125: 33.3 u/ml, cancer antigen 19-9: 12.3 u/ml, and human epididymis protein 4: 63.9 u/ml. The risk of ovarian malignancy algorithm index was 13.2. Transvaginal ultrasonography was not performed because of her virginity. After providing informed consent, the participant opted for surgical excision of the inguinal mass by laparoscopy (Figures 2-3). The inguinal mass was excised by an expert laparoscopist and the diagnosis of endometriosis was established through the final pathology after surgery. The participant was well and has not had any pain during her menstrual cycle since the surgery. The most important reason for reporting this case was that the individual did not have a history of abdominal surgery and was misdiagnosed for four yr (Figure 4).

### Ethical considerations

Written consent was obtained from the participant for reporting the case.

## 3. Discussion

Endometriosis has been shown to be estrogen-dependent but its etiology is multifactorial (1, 2). Retrograde menstruation explained the theory that endometrial cells protrude from the end of the fallopian tubes and the menstrual blood remains on the peritoneal surfaces of the pelvis and abdomen.

Inguinal endometriosis is an uncommon problem that occurs in 0.6% of women (4). Women with inguinal endometriosis have mild to severe dysmenorrhea and a mass in this area (2, 4).

Overall, the proposed hypotheses for the cell origin can be categorized into two main theories: the in-situ theories and the transplantation theories. However, the exact origin and mechanism of endometriosis development remain theoretical (4, 5). The growing body of evidence confirms the multifactorial nature of endometriosis that is the result of the combined contribution of anatomical, hormonal, immunological, reactive, estrogenic, genetic, epigenetic, and environmental factors in affected women (8, 10). Two different theories have been shown: 1) the sigmoid colon supports the left inguinal canal, and 2) endometrial cells remain on the right side because of the clockwise movement of intraperitoneal fluid (10). Our case had right inguinal endometriosis.

Differential diagnosis is necessary in cases of suspected inguinal mass (9). Abdominal sonography, transvaginal sonography, transrectal sonography, magnetic resonance imaging (MRI) and computed tomography (CT) are useful diagnostic instruments. However, if the ultrasound is performed by an expert radiologist, it will also be valuable. The appearance of inguinal endometriosis in a CT scan is often not specific (9); it appears as solid, cystic or complex. An MRI is more accurate than a CT scan. Two MRI patterns are shown for inguinal endometriosis. The type I pattern includes cystic hyper-intense lesions and the type 2 pattern is of lesions with solid components (10). Ultrasound findings of inguinal endometriosis are variable. Cystic masses, represented by intra-cystic bleeding with menstruation, are found in most cases; our participant had an oval lesion. Differential diagnosis in cases of cystic inguinal masses include inguinal hernia and hydrocele, and in cases of solid masses include sarcoma, lymphoma, hematoma and abscess. In inguinal endometriosis, differential diagnosis based on imaging results should be accompanied by a history of dysmenorrhea because of not being of specific diagnostic modality.

Needle aspiration cytology provides accurate diagnosis before removal of malignancy and could be useful in selected cases (9). Women with inguinal endometriosis usually have a history of previous surgery. If inguinal endometriosis is correctly diagnosed before the operation, the woman can undergo laparoscopic surgery to remove the pathology; accordingly, an ultrasound was done for our participant and inguinal endometriosis was reported so she underwent laparoscopic surgery. The participant of our study had no problems except the brief pain at the site of the trocars and was discharged in good general condition the day after the operation.

The pathologic diagnosis was inguinal endometriosis. Therefore, attention to inguinal endometriosis in women with an inguinal mass is important even if there is no history of gynecological or abdominal surgery. Treatment of inguinal endometriosis is surgical removal of the mass (10).

## 4. Conclusion

Considering the possibility of inguinal endometriosis in the differential diagnosis of women with an inguinal mass is important, even if there is no history of gynecologic or abdominal surgery.

##  Conflict of Interest

The authors declare no conflict of interest.
